# Understanding Miro GTPases: Implications in the Treatment of Neurodegenerative Disorders

**DOI:** 10.1007/s12035-018-0927-x

**Published:** 2018-02-06

**Authors:** Laura Kay, Ilse S. Pienaar, Ruwini Cooray, Gary Black, Meera Soundararajan

**Affiliations:** 1 Department of Applied Sciences, Faculty of Health and Life Sciences, Northumbria University, Newcastle, NE1 8ST UK; 2 School of Life Sciences, University of Sussex, Falmer, BN1 9PH UK

**Keywords:** Miro GTPase, Neurodegenerative disease, Parkinson’s disease, Mitochondria, Atypical GTPase, Amyotrophic lateral sclerosis, Alzheimer’s disease

## Abstract

The Miro GTPases represent an unusual subgroup of the Ras superfamily and have recently emerged as important mediators of mitochondrial dynamics and for maintaining neuronal health. It is now well-established that these enzymes act as essential components of a Ca^2+^-sensitive motor complex, facilitating the transport of mitochondria along microtubules in several cell types, including dopaminergic neurons. The Miros appear to be critical for both anterograde and retrograde mitochondrial transport in axons and dendrites, both of which are considered essential for neuronal health. Furthermore, the Miros may be significantly involved in the development of several serious pathological processes, including the development of neurodegenerative and psychiatric disorders. In this review, we discuss the molecular structure and known mitochondrial functions of the Miro GTPases in humans and other organisms, in the context of neurodegenerative disease. Finally, we consider the potential human Miros hold as novel therapeutic targets for the treatment of such disease.

## The Miro GTPases: Regulators of Mitochondrial Function

Mitochondria are essential for energy production, neuronal function, cellular survival and control of intracellular calcium homeostasis [1, 2]. Mitochondrial ATP production supports essential neurological functions including generation of membrane potential, spike potential, mobilisation of synaptic vesicles and mediating presynaptic development [3, 4]. In addition, mitochondria play a critical role in mediating calcium homeostasis during neuronal stimulation and regulate neurotransmission and short-term plasticity [5–8]. In order to maintain this plethora of functions including mitochondrial movement, morphology, fission, fusion and ATP production, the individual processes need to be very finely regulated. Two different mitochondrial Rho GTPases, Miro1 and Miro2, have been shown to play an intricate role in all of these mitochondrial functions. Since abnormalities in mitochondrial function strongly associate with various neurological dysfunctions, understanding the role of Miro1 and Miro2 remains vital to understanding several human neuropathologies.

Initially classified as typical Rho GTPases [9], the Miro GTPases (‘mitochondrial Rho GTPases’) are now considered a subclass of the Ras monomeric GTPase superfamily [10–12]. The Miro subfamily contains only the two genes encoding the Miro GTPases present in humans, namely Miro1 and Miro2 (alternatively referred to as RhoT1 and RhoT2) [9]. Anchored to the mitochondrial outer membrane (MOM) by means of a C-terminal transmembrane sequence [12, 13], the Miro proteins are accessible to the cytoplasm, where they are involved in a variety of mitochondria-related processes, including the morphology and homeostasis, anterograde and retrograde movement of mitochondria [14–18]. Considering the importance of healthy mitochondria in neuronal function, in addition to the strong implication of dysfunctional mitochondria in psychiatric disorders such as schizophrenia and neurodegenerative conditions such as Alzheimer’s disease (AD) and Parkinson’s disease (PD) [7, 19–22], a comprehensive understanding of the Miro proteins holds significant clinical importance.

## Molecular Structure of the Miro GTPases

**Fig. 1:**
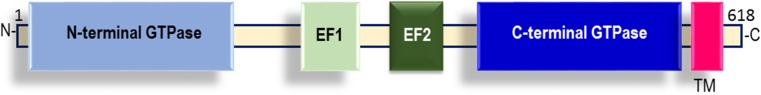
Domain architecture of human Miro GTPase. The amino terminal and carboxy terminal GTPase domains are shown in light and dark blue, respectively. The two calcium binding EF-hand domains, flanked by the catalytic domains, are depicted in light and dark green. The extreme C-terminal transmembrane domain responsible for anchoring mitochondria to the outer membrane is shown as a pink rectangle

The Miro GTPases are the only human proteins comprising two different GTPase domains in the same polypeptide chain. A pair of Ca^2+^ binding helix-loop-helix topology containing EF-hand domains is flanked by the two GTPase domains [9, 11, 13, 14, 23–29] (Fig. 1). Both human Miros consist of 618 amino acid residues, sharing 60% amino acid identity between them [9, 13, 27]. The Miro GTPases contain a transmembrane domain required for targeting to the MOM where they are anchored at the C-terminus, with the majority of the protein accessible to the cytoplasm [12, 13, 30, 31] (Fig. 1). Of these GTPase domains, the N-terminal GTPase domain is the most well-studied and, structurally, most similar to Rho GTPases [27]. Conversely, the C-terminal GTPase domain appears similar to Rheb, a protein of the Ras sub-family by sequence homology [12, 13].

Despite the N-terminal GTPase domain’s similarity to Rho GTPases, initial classification of the Miro GTPases as members of the Rho sub-family was disregarded when both GTPase domains were found to lack the conserved G-3 DxxG motif [17, 32–34] and Rho-specific insert helix [18, 27, 35, 36], a surface-exposed alpha-helical domain unique to the Rho GTPases. The two EF-hand domains of Miro have been shown to bind Ca^2+^ [16, 23] and the bordering regions of these domains appear highly conserved amongst eukaryotes [23, 24]. A recent crystallographic study showed that these bordering regions contain non-canonical ‘hidden’ EF-hands (hEF-hands) proceeded by single helices (LM helices 1 and 2) in the single drosophila Miro (dMiro) orthologue [23]. In dMiro, these hEF-hands exhibit a helix-loop-helix structure capable of stabilising local EF-hands via formation of an anti-parallel EF-hand β-scaffold. The structure of the LM helices, however, is similar to that of extrinsic ligands bound to EF-hand proteins, as described for the protein complexes of moluscan myosin heavy and light chain [23, 29], as well as troponin I and troponin C [37]. These structural features are shown to be highly conserved in human Miro x-ray crystallographic structures reported recently [38]. This combination of EF-hEF hands followed by an LM helix has been observed in a variety of other Ca^2+^ binding proteins, including recoverin, the neuronal calcium binding protein found in photoreceptor [24, 35, 39, 40], the pollen protein polcalcin which is implicated as a panallergen [41] and a human guanylate cyclase-activating protein (GCAP3), a calcium-dependent guanylate cyclase receptor in phototransduction pathways [25, 36, 41]. The domain architecture and subatomic structures of the Miros suggests that these proteins have structurally and functionally evolved to cater as GTPases, with an N-terminal domain and unique ‘putative catalytic domain’ in the C-terminus, in addition to modified EF-hands that can function as calcium sensors.

## Miro Facilitates Mitochondrial Transport

A well-documented function of Miros is the central role they play in the transport of mitochondria [9, 11, 13–18, 42, 43], facilitated by the action of kinesins and dyneins acting as anterograde and retrograde motors, respectively [44]. In some motor neurodegenerative disorders, deficiencies in mitochondrial transport are most notable in neuronal cells, where efficient transport of mitochondria from the nucleus to components with high energy demands, such as synaptic terminals, is critical for healthy neuronal function and survival [45]. However, retrograde mitochondrial movement also appears crucial in neuronal health [46].

**Fig. 2:**
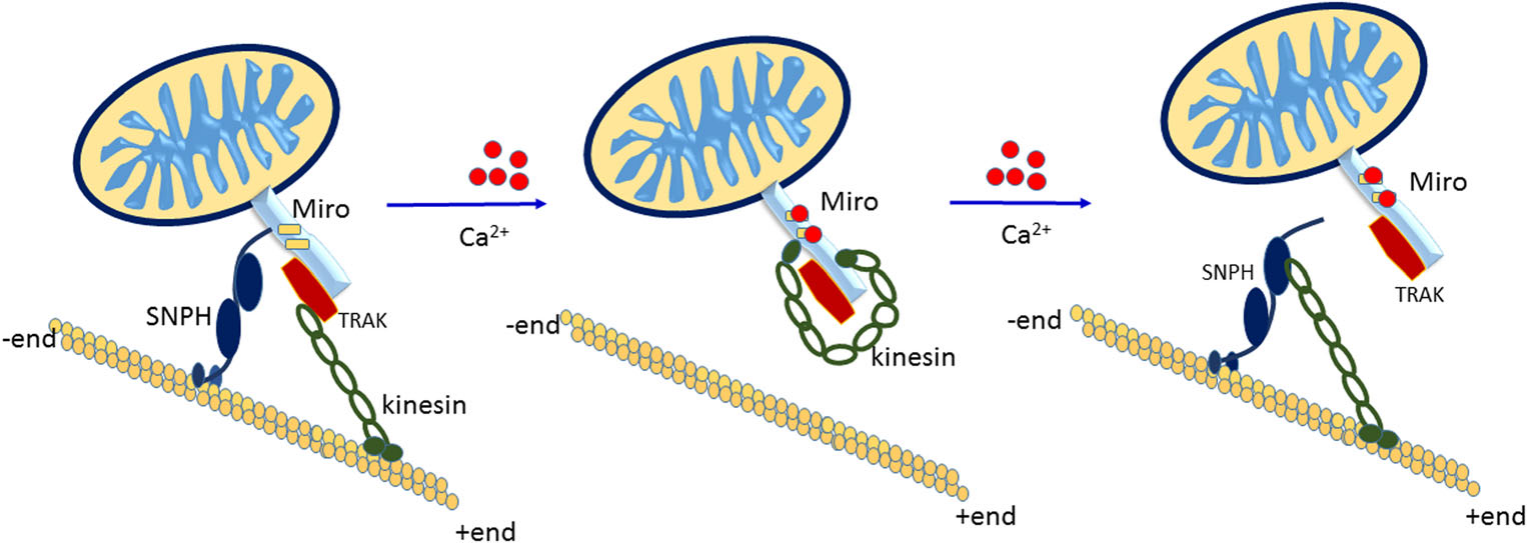
Miros in mitochondrial movement. The Miros act within an integrated machinery with TRAK1/2 to facilitate the anterograde and retrograde movement of mitochondria along microtubules. Both axonal and dendritic mitochondrial transport are mediated by the Miros, although they appear to engage different transport machineries to achieve this. TRAK1 binds to both kinesin-1 and dynein/dynactin and is predominantly localised in axons, while TRAK2 preferentially binds dynein/dynactin and exhibits dendritic localization. The interaction of TRAK1 with both the kinesin (anterograde) and dynein (retrograde) motors enable movement in both directions in the axon, while TRAK2’s more favourable interactions with dynein may encourage retrograde movement at neurons’ distal ends. Only the anterograde movement is shown in this figure. Miro EF-hands are represented by yellow rectangles; calcium is represented by red spheres. The molecules and mitochondria are not depicted to scale

A search for genes necessary for axonal and synaptic function in *Drosophila melanogaster* revealed key roles for dMiro in the transport of mitochondria from the neuronal soma to distal synapses [14]. Glater and colleagues reported that a protein complex composed of dMiro and the kinesin-associated protein Milton enable the anterograde transport of mitochondria via apparent recruitment of kinesins [47]. Two mammalian homologues of Milton, trafficking kinesin-binding protein 1 (TRAK1) (also known as OIP106) and trafficking kinesin-binding protein 2 (TRAK2) (also known as OIP98/Grif-1), capable of forming complexes with mammalian Miro1 and Miro2 and with microtubule motors, have also been shown to co-localise with human Miros (hMiros) [15], indicating that these proteins act as a component of a conserved protein complex necessary for mitochondrial transport (Fig. 2).

The anterograde motor kinesin-1 (also referred to as kinesin heavy chain (Kif5)) and the retrograde motor (the dynein/dynactin complex) were shown to facilitate the transport of many cellular cargoes along microtubules [48]. These motor proteins are bound to mitochondria by interacting with two mitochondria-specific proteins: Miro and Milton (or the Milton homologues TRAK1 and TRAK2 in mammals). Miro anchors to the mitochondrial outer membrane while Milton serves as an adaptor protein, linking the motor proteins to Miro and therefore to mitochondria. The resulting protein complex is believed to facilitate the movement of mitochondria along microtubules [13, 15–17].

Interestingly, while the concept of the Miro/Milton (TRAK) transport complex is widely accepted, direct (Ca^2+^-dependent) binding of hMiro1 to kinesin motor Kif5 has been demonstrated, indicating a degree of redundancy for a Milton-like adaptor protein [16]. In contrast, TRAK2 and hMiro1 have been shown to directly form a protein complex and co-localise with mitochondria in mammalian brain tissue extracts [15]. Furthermore, the GTPase state of the hMiro1 N-terminal GTPase domain appears to recruit TRAK2 to mitochondria in mammalian cell lines, producing downstream effects on anterograde mitochondrial movement [15]. Indeed, over-expression of hMiro1 appears to increase TRAK2 recruitment to mitochondria that, in turn, encourages anterograde mitochondrial transport. Correspondingly, abolishing the kinesin-binding domain in TRAK2 impairs anterograde movement of mitochondria [15]. This suggests that transport of mitochondria in mammals is mediated by a mechanism dependent on the N-terminal GTPase domain for recruitment of TRAK to the mitochondria and that the resulting Miro-TRAK-kinesin protein complex is required for anterograde movement of mitochondria along microtubules. However, retrograde mitochondrial movement may also be affected by aberrant Miro function, with recent live-imaging of GFP-tagged mitochondria showing that dysfunctional dMiro results in the impairment of both anterograde and retrograde mitochondrial transport [17]. Indeed, both Miro1 and Miro2 coupled with the disrupted in schizophrenia 1 (DISC1) protein, influencing the mitochondrial transport and fusion machinery via the TRAK1 and TRAK2 molecular adapters [49]. Analysing the role of DISC1 and proteins associated with mitochondrial dynamics has recently revealed that disruption of the DISC1 Miro/TRAK complex inhibits mitochondrial transport in neurons [49, 50]. Characterisation revealed that the Miro-DISC complex acts as a regulatory unit in mediating mitochondrial dynamics in both axons and dendrites [49, 50]. This is of note since it provides compelling evidence that the Miro-TRAK complex can play a role not just in axons, as previously shown, but also in dendritic mitochondrial trafficking [50, 51].

Despite the suggestion that a Milton/TRAK adaptor could be redundant under some disease conditions due to direct Miro-kinesin motor binding [16], both TRAK1 and TRAK2 have been directly linked to mitochondrial motility [13, 15–17]. Indeed, recent studies suggest a link between nutrients available to neurons and mitochondrial motility through glucose signalling and subsequent modification of TRAK1 and 2 [52, 53]. In this regard, extracellular glucose was shown to activate O-GlcNAc transferase (OGT), an enzyme that catalyses post-translational O-glycosylation of target proteins [52]. Proteomic investigations suggest that activated OGT targets TRAKs for GlcNAcylation, leading to the arrest of mitochondrial motility [53].

Taken together, accumulating evidence suggests that a Miro/Milton (TRAK)/motor transport complex is involved in the transportation and motility of mitochondria and that this is sensitive to signalling from within the complex [15, 16], from intracellular components [23, 50] and from external factors such as extracellular glucose levels [52, 53]. Interestingly a very recent study from Melkov and others [54] presents an alternative model for mitochondrial transport by Miro-based motor complex where they differentiated the mitochondrial anterograde and retrograde movement using *Drosophila* bristle cells that mimic neurons. Here, they show through a microtubule gliding assay the dynein-mediated bidirectional mitochondrial transport was mediated by Miro in retrograde mitochondrial transport while Milton was observed to be responsible for primary polarised mitochondrial sorting into the developing bristle cells [54]. The study shows lymphocyte mitochondria specifically redistribute to the adhesion zone in close contact with the endothelium. Miro-1, through the regulation of mitochondrial movement along microtubules and its association with dynein/dynactin motors, influences mitochondrial positioning. Deficiency in Miro-1 prevents correct interaction with inflamed endothelium, lymphocyte polarisation and chemotactic migration.

## The Miro GTPases Facilitate Ca^2+^-dependent Transport of Mitochondria

While the necessity of the Miro/TRAK/motor complex in mitochondrial transport is widely accepted, the role of cytosolic Ca^2+^ in relation to this complex remains disputed. Cytosolic Ca^2+^ is required for mitochondrial transport, which is arrested in the presence of increased intracellular Ca^2+^ [55]. Interestingly, the Miro EF-hands are not only involved in binding calcium [56], but in sensing the influx of Ca^2+^ during synaptic activation, triggering conformational changes in Miro to regulate the protein-protein complexes and binding of effector molecules through the N-terminal GTPase domain effector loop [57]. This is crucial since Ca^2+^ sensing and the regulation of intermolecular interaction dictates mitochondrial immobilisation at active synapses [16, 18, 57]. Various predictive models have been proposed regarding the link between Miro, Ca^2+^, and mitochondrial transport (Fig. 2). One model stipulates that increased cytosolic Ca^2+^ initiates dissociation of the kinesin motor from microtubules and that the subsequent interaction of kinesin with Miro on the mitochondrion results in the dissociation of motors from the microtubules (Fig. 2). An alternative model suggests that Miro binds directly to kinesin without the need for the Milton adaptor, and that increased cytosolic Ca^2+^ inhibits Miro’s interaction with kinesin, leading to direct uncoupling of Miro from kinesin [16] (Fig. 2). However, the arrest of mitochondrial transport in neurons has also been linked to the mitochondrial tethering protein syntaphilin (SNPH), resulting in a third model being proposed. In this so-called Engine-Switch and Brake model, increased cytoplasmic Ca^2+^ dissociates kinesin from Miro [58, 59] (Fig. 2). Following dissociation, kinesin then interacts with SNPH, disrupting the ATPase activity of kinesin and resulting in the arrest of mitochondrial motility. Thus, SNPH performs as an ‘engine-off’ switch by detecting Ca^2+^-induced arrest of mitochondria, and also as a brake, by securing static mitochondria to the microtubules.

An alternative proposition holds that intra-mitochondrial Ca^2+^, rather than cytosolic Ca^2+^, plays a critical role in mediating mitochondrial transport, and that Miro is involved in orchestrating intra-mitochondrial Ca^2+^ levels [59]. Mitochondria buffer cytoplasmic Ca^2+^ via uptake of Ca^2+^ through the mitochondrial calcium uniporter (MCU) [60]. The uptake of Ca^2+^ through the MCU was shown to be inversely related to mitochondrial velocity in axons, thus illuminating a mechanism by which cytosolic Ca^2+^ influences mitochondrial trafficking [59]. Two independent studies have demonstrated that expression of Miro1 at the mitochondrial outer membrane affects the concentration of Ca^2+^ in the mitochondrial matrix [18, 59]. As elevated intra-mitochondrial Ca^2+^ has been associated with slower movement or stopping the movement of mitochondria, alongside a subsequent increase in ATP production, these results indicate that a link exists between mitochondrial motor machinery, mitochondrial trafficking and the mediation of bioenergetic efficiency in mitochondria [61, 62].

## The Miro GTPases in Mitochondrial Morphology

The influence of Miro on mitochondrial morphology appears to be strongly conserved. Initial functional studies in mammalian cells showed perinuclear aggregation of mitochondria when a mutant of hMiro1 bearing a constitutively active N-terminal GTPase domain was over-expressed [9]. A similar effect was obtained from over-expression of a mutant of hMiro1 harbouring a dominant-negative N-terminal GTPase domain, though to a lesser extent [9]. These results imply that a balanced level of Miro activity in the N-terminal GTPase domain of hMiros is necessary for the maintenance of normal mitochondrial morphology. The single Miro protein of *Saccharomyces cerevisiae*, Gem1p, appears to require both GTPase domains and functional EF-hands for the maintenance of normal mitochondrial morphology [11]. A 662-amino acid protein, Gem1p shares 30% amino acid identity with the human Miros. When Gem1p was ablated in *S. cerevisiae* (Gem1pΔ cells), mitochondria exhibited both abnormal distribution and abnormal morphology, with a collapsed, globular or ‘grape-like’ appearance [28]; however, such mitochondria retained inner cristae structures when viewed under transmission electron microscopy.

A role for Miro in mitochondrial morphology has also been observed in *Drosophila*, with overexpression of wild-type dMiro producing significant aggregation of mitochondria in dopaminergic neurons [43] and abnormally elongated mitochondria in larval motor neurons [17, 43]. However, the effects of dMiro on mitochondrial morphology may be dependent on context and cell type in vivo; if so, this would suggest that dMiro is not directly involved in modulating mitochondrial morphology but perhaps that one or more binding partners are necessary to exert the effects on mitochondrial morphology observed previously [9, 17, 63].

Early research on the human Miros [9, 27] concentrated on the creation of Miro mutants containing amino acid substitutions in the N-terminal GTPase domain, making this GTPase domain either constitutively active (G13V) or dominant negative (S18N) with respect to GTP/GDP-bound status. Ectopic expression of Miro1 mutants bearing the constitutively active N-terminal GTPase domain (Miro1 V13) induced a collapse of the mitochondrial network in non-neuronal cells, with mitochondria exhibiting perinuclear aggregation [9]. Ectopic expression of this mutant was associated with increased presence of the apoptotic marker M30 (recognising caspase-cleaved cytoskeleton-18) relative to both cells expressing wild-type Miro1 and cells ectopically expressing S18N Miro1 mutants. Correspondingly, the introduction of caspase inhibitors reduced this increase in M30, suggesting a role for the GTP/GDP-bound status of the Miro1 N-terminal GTPase domain in apoptosis. However, while overexpression of Miro in other organisms has produced a similar pattern of mitochondrial aggregation, other studies have failed to demonstrate a clear link between Miro overexpression and apoptosis [9, 27].

## The Miro GTPases in Mitochondrial Fission and Fusion

**Fig. 3:**
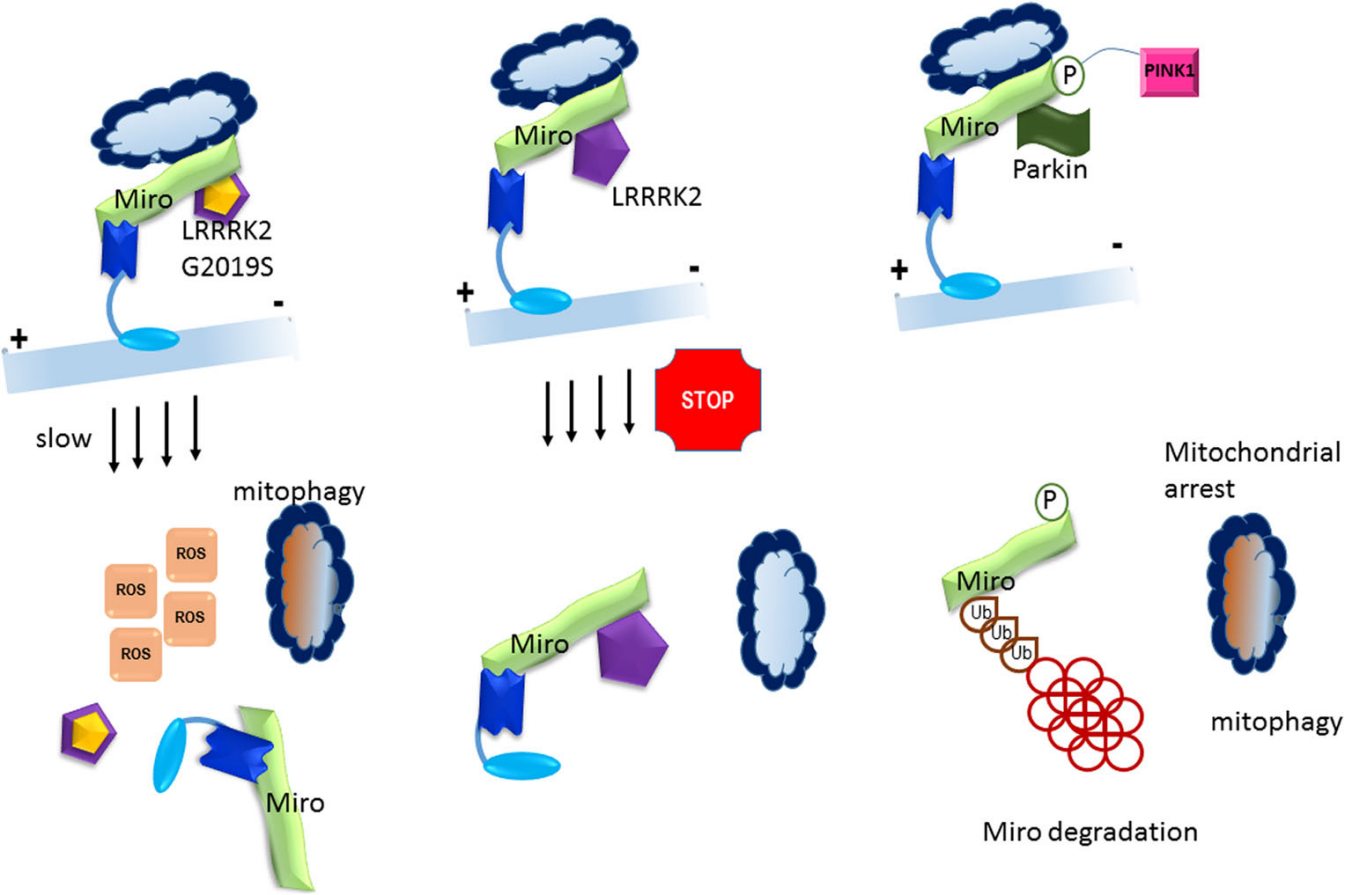
Miro in Parkinson’s disease. A = LRRK2, promotes Miro removal by forming a complex with Miro. Pathogenic mutant LRRK2G2019S disrupts this function, delaying the arrest of damaged mitochondria and consequently slowing the initiation of mitophagy. Mitochondrial motility and Miro degradation are shown to be impaired in PD patients. Direct interaction of LRRK2 with Miro results in Miro removal from mitochondria. In pathogenic LRRK2 mutant G2019S this is deranged delaying the arrest of damaged mitochondria and consequently slowing the initiation of mitophagy. Knockdown in Miro levels in *LRRK2G2019S* human neuron and *Drosophila* PD models rescued neurodegeneration. Miro degradation and mitochondrial motility are also impaired in sporadic PD patients. B = Parkinson’s disease implicated PINK1 kinase and Parkin play an important role in quality control of mitochondrial survival and apoptosis through Miro GTPase. Dysfunctional mitochondria are destroyed after PINK1 accumulation that phosphorylated Miro at S156 and also Parkin to activate its E3 ligase activity. This results in proteosomal degradation of Miro and mitochondrial arrest and mitophagy

Mitochondrial fission, fusion, and transport play important roles in healthy mitochondrial network [1, 2, 64]. The balance between fusion and fission controls mitochondrial morphology, which is mediated by a number of enzymes including the Miro GTPases (see Fig. 3). A recent effort to identify regulators of Miro identified that Vimar in *Drosophila*, and its mammalian homologues RAP1GDS1, regulated mitochondrial morphology. The Vimar homologues function as guanine nucleotide exchange factor (GEF) proteins, regulating mitochondrial fission in response to calcium concentrations. Under normal cellular conditions, Miro increases mitochondrial size by inhibiting Drp1 [43, 63]; however, at high concentrations of Ca^2+^, Miro interacts directly with Vimar homologues and promotes mitochondrial fission [65]. The mitochondrial enlargement observed in the *Drosophila* model PD was rescued through loss of Vimar expression [65]. RAP1GDS, the mammalian homologue of Vimar, exhibits the conserved biological function seen in *Drosophila*. Targeting the human Miro/RAP1GDS1 complex through peptides or small molecule drugs may therefore prove a promising therapeutic approach, avoiding any off-target effects that could occur by singling out individual molecules as targets.

## Effects on ATP Homeostasis

While Miro 1 appears abundantly expressed in heart and skeletal muscle, Miro 2 expression is most prominent in heart, liver, kidney, pancreas and skeletal muscle tissue [9]. This is particularly interesting with regards to the high energy demand these cell types commandeer, suggesting perhaps that the Miro GTPases are involved in ATP homeostasis or cellular bioenergy homeostasis. Indeed, a Gem1p abrogation strain in *S. cerevisiae* grew significantly slower on glycerol minimal media relative to wild type cells, suggesting that yeast Miro homologue Gem1p is necessary for correct mitochondrial respiration [11]. Too, the single Miro homologue GemA in *Dictyostelium discoideum* appears to play a role in mitochondrial respiration, with GemA knockout mutants exhibiting impaired cell growth on nutrient media alongside reduced ATP content and increased oxygen consumption [24]. This function of Miros in maintaining ATP homeostasis is likely to be conserved across species.

## Animal Models of Miro Abrogation

The function of the Miro enzymes has been evaluated in several different model organisms. Most closely related to Miro1 in humans, dMiro represents a single Miro protein expressed in *Drosophila*. Mitochondria in mutant dMiro neurons was adversely altered relative to wild-type controls, with neat clustering of mitochondria observed towards the soma of mutant larval neurons and an absence of mitochondria noted at distal neuronal structures, such as neuromuscular junctions [14]. *Drosophila* mutant larvae presented with a slim body relative to wild type, with abnormally small muscle size and progressive locomotive defects including increasing levels of paralysis, culminating in death at either the larval or early pupal stage. This phenotype was rescued by expressing wild-type dMiro in neurons, but not in muscle cells, suggesting a critical role for dMiro in neuronal function and survival [14]. Interestingly, mutations affecting axonal transport often present with abnormal pre-synaptic vesicle accumulation, and typically, this state of accumulation is a marker of neuropathology [66–68]. While vesicular transport appeared impaired in dMiro mutant neurons, however, this effect was qualitatively and quantitatively diverse from the significant defects in mitochondrial transport observed [14]. Thus, while the impairment of vesicular transport may have contributed to the observed dMiro mutant phenotype, it is unlikely that both transport defects were the consequence of a shared mechanism.

Other than the dMiro mutant flies, recent global and neuron-specific Miro1 mouse knockouts have been developed [69]. Mice globally deficient of Miro1 were cyanotic and died shortly following birth. The Miro1 neuron-specific knockout mouse phenotype was also striking, exhibiting rapidly progressing upper motor neuron disease symptoms and early death after approximately 4 weeks. At birth, the neuron-specific Miro1^−/−^ mice appeared indistinguishable from WT littermates. However, by 2 weeks, Miro1^−/−^ mice exhibited hind-limb clasping, a known early marker for neuronal impairment. These mice failed to gain weight as they matured and developed a stiff tail, spinal curvature (kyphosis), hind-limb spasticity, and progressive locomotive defects. This phenotype was reflective of the development of upper human amyotrophic lateral sclerosis (ALS), with symptoms becoming progressively worse and premature death occurring at approximately 35 days. Impaired retrograde transport of mitochondria was implicated in the development of this phenotype, rather than the anterograde transport impairment strongly implicated in earlier studies. However, the previously observed perinuclear aggregation of mitochondria was shown in mouse embryonic fibroblasts obtained from Miro1^−/−^ mice [69]. No significant differences were reported in mitochondrial respiration or mitochondrial membrane potential in Miro1^−/−^ cells relative to controls, indicating that defective mitochondrial transport was the primary cause of the mutant phenotypes, but that this transport was not influenced by defective mitochondrial respiration or membrane potential.

## The Miro GTPases in Neuronal Pathology

The Miro GTPases appear to play a critical role in the maintenance of neuronal health. This is perhaps unsurprising when one considers the crucial role the Miros appear to play in mitochondrial transport (discussed previously) coupled with the need for mitochondria to travel vast distances in neurons along axons (which can be up to ~ 1 m long) from the soma towards the distal synaptic end for neural transmission [70–72]. Indeed, altered Miro function has been associated with CNS pathologies such as PD [43, 73], and ALS [14, 69, 74]. Moreover, disruption of mitochondrial dynamics by targeting the DISC1-Miro/TRAK complex or upon expression of the DISC1-Boymaw fusion protein impairs the correct development of neuronal dendrites [51].

## The Role of the Miros in Development of Upper Motor Neuron Disorders

An investigation into the role of the Miros in upper motor neuron development and mitochondrial retrograde transport using mouse knockouts (KO) clearly demonstrated a compelling role for Miro1 in neurological disorders through its influence of mitochondrial motility [69]. In this study, Nguyen and colleagues showed that the Miro1 mouse KO clearly displayed physical hallmarks of neurological disease in the brainstem and spinal cord [69]. The mice developed rapidly progressing upper motor neuron disease symptoms in 4 weeks. The role of Miro in mitochondrial motility is therefore worthy of consideration, as the defects in mitochondrial motility caused by Miro1 was shown to be sufficient to cause progressive MND. Hence, a complete understanding of the causal and possible therapeutic role of Miros in upper MNDs such as spinal cord injury, cerebral palsy, multiple sclerosis and acquired brain injury including stroke remains to be established.

## Miro Proteins in Parkinson’s Disease

It is estimated about 10 million people live with PD worldwide and approximately 60,000 Americans are diagnosed with PD every year [75]. Although PD is typically diagnosed in individuals above age 65 [76], diagnosis in patients below age 65 is increasing [77]. In 2010, the total cost of PD was €13.9 billion in Europe [78] and $14.4 billion in the USA [79], with costs projected to progressively increase [79, 80]. PD is characterised by the degeneration of dopaminergic neurons and/or loss of neuronal projections in several dopaminergic networks [81]. Current treatments for idiopathic PD rely mainly on the use of pharmacologic agents to improve disease symptoms [82]. Since PD remains an incurable disease, it is crucial to establish new therapeutic strategies for PD treatment. It is therefore of great clinical interest to identify PD biomarkers and validate novel drug targets with the ageing population increasing worldwide every year.

Interestingly, PD has been consistently associated with mitochondrial dysfunction [83, 84]. Indeed, several reliable PD animal models rely on exposure to mitochondria toxins, such as MPTP [85] and rotenone [86]. Furthermore, some monogenetic forms of PD are mitochondria-related. For example, mutations in leucine-rich repeat kinase 2 (LRRK2) are associated with autosomal dominant PD [87] and have been implicated in mitochondrial fragmentation and increased apoptotic rates relative to wild-type LRRK2 [88–90].

(see Fig. 3). Moreover, LRRK2 has been shown to partially co-localise with mitochondrial fission dynamin-like protein 1 (DLP1) in cortical neurons, suggesting that pathogenic LRRK2 mutants may be associated with PD through disturbances in mitochondrial fission [90]. As discussed below, mutations in the PTEN-induced putative kinase 1 (PINK1) and E3 ubiquitin ligase Parkin have also been linked to mitochondrial-related autosomal recessive manifestations of PD [91].

In *Drosophila*, overexpression of dMiro has been demonstrated as toxic, producing an age-dependent loss of dopaminergic (DA) neurons, the neurons specifically affected in the substantia nigra of PD patients [92, 93]. The exact process by which this dMiro overexpression produces toxicity remains obscure. However, Miro GTPases are known to be associated with proteins involved with PD when dysfunctional: the mitochondria-localised PINK1 and Parkin, an E3 ubiquitin ligase usually localised in the cytoplasm [94]. Under normal circumstances, PINK1 and Parkin proteins form crucial components of a mitochondrial quality control system aimed at targeting damaged mitochondria for isolation and mitophagy, in order to sustain cellular metabolic requirements and prevent damage caused by defective mitochondria [95, 96]. Loss-of-function mutations in PINK1 and Parkin are associated with rare recessive forms of PD [97]. Mitochondrial damage results in PINK1 accumulation on the outer mitochondrial membrane (OMM) and recruitment of Parkin from the cytosol to mitochondria. Upon recruitment, Parkin ubiquitinates various substrates on the OMM [VDAC1, dynamin-related protein 1 (Drp1), Mfns, translocase of outer mitochondrial membrane 20 (TOM20) and TOM40], allowing for initiation of mitophagy by the ubiquitin/proteasome pathway [98, 99]. Interestingly, Miro appears to interact with PINK1 and Parkin and is ultimately targeted for ubiquitination by Parkin when mitochondrial damage occurs. In a *Drosophila* PD model involving loss of PINK1 function, reduced dMiro function improved the degenerative phenotype shown in PINK1 mutant DA neurons. This suggests a role for mitochondrial transport and Miro in PINK1-related PD pathogenesis [92], a notion further supported by the profound effects seen in altered PINK1 function on the transportation of axonal mitochondria in *Drosophila* larval motor neurons or mammalian hippocampal neurons. Indeed, Miro appears to be specifically targeted for degradation by PINK1 and Parkin in vivo in *Drosophila* or in cultured mammalian cells treated with the mitochondrial toxin carbonyl cyanide m-chlorophenylhydrazone (CCCP) [92, 100, 101]. Whether Miro is a direct substrate of PINK1-mediated phosphorylation or whether this phosphorylation is a prerequisite for the regulation of Miro stability by PINK1 and Parkin remains unknown [92, 102]. Miro has previously been shown to undergo PINK1-mediated phosphorylation at Ser156 and that phosphorylation at this site is necessary for degradation of Miro by Parkin [73]. The loss of hMiro in HeLa cells resulted in the perinuclear aggregation of mitochondria and facilitated in increased mitophagy, a phenotype previously associated with activation of the PINK1/parkin pathway [92]. It has also been postulated that Miro may form a constituent of the Parkin receptor complex, as hMiro1 appears capable of stabilising phospho-mutant versions of Parkin on the OMM. The regulation of Miro stability and turnover by PINK1 and Parkin could perhaps act to isolate damaged mitochondria from the network, promoting their transport to the cell body and subsequent degradation. However, further studies are required to elucidate the underlying molecular interplay between Miro, PINK1 and Parkin using PD patient samples.

## Miro Proteins in ALS

ALS, also known as motor neuron disease, is characterised by progressive upper and lower motor neuron degeneration, resulting in severe limb and trunk muscle weakness, and eventual paralysis [103]. Several studies have described altered expression levels and/or dysfunctional Miro in ALS patients or animal models of the disease. This included a report of significantly reduced levels of Miro1 present in spinal cord samples of ALS patients [104]. In addition, the same group found that protein levels were also depleted in an experimental model of the disease, using transgenic mice expressing familial ALS-associated mutations in genes encoding copper-zinc superoxide disputes 1 (SOD1) G93A or TAR DNA-binding protein 43 (TDP-43) M337V, with these mutant mice displaying a phenotype that closely resembles clinical ALS. In the transgenic mice, the Miro1 protein levels were found to be reduced exclusively in the spinal cord, and not in brain tissue, potentially explaining the selective vulnerability of motor neurons in the spinal cord during ALS. The authors concluded that the Miro1 deficiency observed in this study may explain the impaired intracellular distribution of mitochondria seen in ALS [105].

Mutations in SOD1 have previously been shown to impair axonal transport of mitochondria in motor neurons isolated from SOD1 G93A transgenic mice, similar to what is seen in ALS-associated mutant SOD1 transfected cortical neurons [106]. A recent investigation by Moller and colleagues [107] revealed the mechanism underlying dysfunctional axonal transport of mitochondria in mutant SOD1-related ALS. The study found that the expression of ALS-related mutant SOD1 reduced the level of endogenous Miro1, and that such reductions were dependent on an E3 ubiquitin-ligase Parkin, which acts downstream of the Ser/Thr-kinase, PINK1. The PINK1/Parkin pathway quarantines damaged mitochondria prior to their clearance through the phosphorylation of Miro by PINK1, which instigates Parkin-dependent ubiquination, and thus the degradation of Miro1, to consequently halt mitochondrial transport in axons [73]. However, another study failed to observe PINK1-dependent Miro phosphorylation, and also could not validate the requirement of Miro’s phosphorylation for subsequent degradation [43]. Yet, the study by Moller and others [107] provided evidence for a PINK1-Parkin-dependent mechanism underlying Miro1 degradation, with the additional finding that expression of ALS mutant SOD1 inhibits axonal transport of mitochondria by activating this pathway.

Calcium binding to Miro1 has been shown to halt anterograde mitochondrial axonal transport by modifying Miro1’s interaction with the motor domain of kinesin-1 via an adaptor protein, Milton. On the other hand, it’s been shown that the EF hand motif of Miro can mediate Ca^2+^-dependent arrest of both retrograde and anterograde motion of mitochondria [57]. Interestingly, the study by Moller and others [107] did not detect changes to cytosolic calcium (Ca^2+^) levels in ALS mutant SOD1-transfected cortical neurons.

Nguyen and others [69] introduced two novel mouse models, created through neuron-specific (corticospinal tract axons) knockout of Miro1 that demonstrated the importance of Miro1-mediated mitochondrial motility and distribution for maintaining neuronal functions. The study further revealed a specific requirement for *Miro1* in upper motor neuron development and post-mitotic function, with targeted disruption of *Miro1* within the cerebral cortex that caused retrograde mitochondrial motility defects in cortical neurons, depletion of mitochondria from neuronal axons within the corticospinal tract, and progressive upper-body ALS. However, despite the negative effects that loss of mammalian Miro1 function exerted on mitochondrial distribution, the loss did not disrupt calcium-regulated mitochondrial movement, mitochondrial-mediated calcium buffering, nor mitochondrial respiratory function. This suggests that defects in mitochondrial motility and distribution are sufficient to cause neurological disease, such as ALS.

## Miro Proteins in Alzheimer’s Disease

**Fig. 4:**
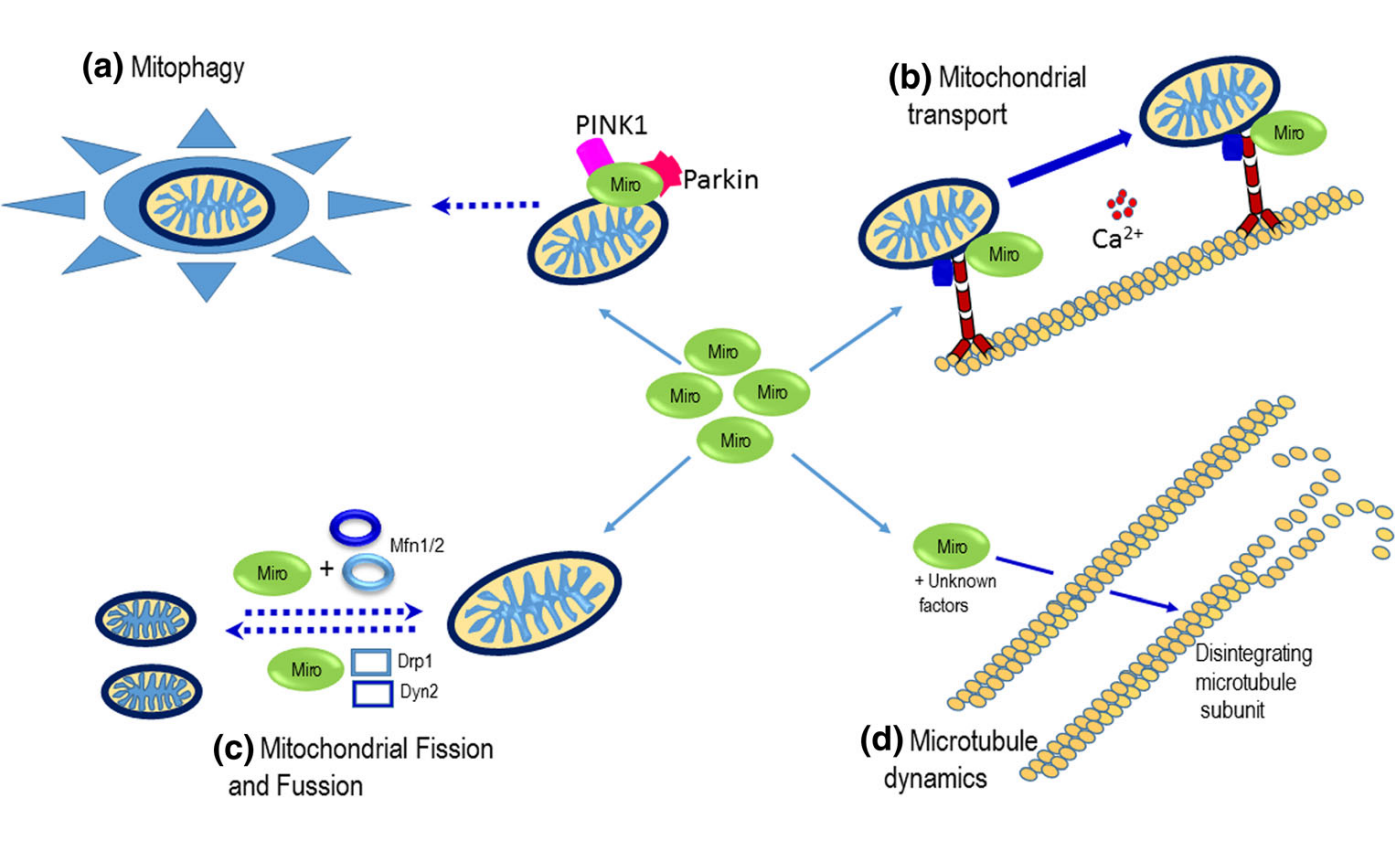
A schematic diagram showing cellular functions of Miro GTPases. A = PINK1 kinase phosphorylates Miro and Parkin subsequently ubiquitinates Miro for proteosomal degradation, which interferes with mitochondrial movement. This process is considered to be a prelude to mitophagy, a process during which damaged mitochondrial are removed. B = The Miros are responsible for mitochondrial transport in the anterograde and retrograde transport in response to energy demand and calcium concentration. C = The Miros play a significant role in maintaining mitochondrial morphology. Mitochondrial morphology is determined by a dynamic equilibrium between organelle fusion and fission. The processes of mitochondrial fission and fusion are also mediated by Miro with other GTPases like mitofusins. D = Microtubule dynamics form ordered cytoskeletal structures that contribute to neuronal polarity maintenance, neuronal morphology and the transportation of cargo. Miro affects microtubule dynamics through an unknown mechanism that may affect cell cycle and cell division in neuronal systems [127]. Mitochondria, microtubule subunits and signalling molecules are not drawn to scale.

Beyond PD, altered Miro function has been implicated in the pathogenesis of other neurological disorders featuring abnormal mitochondrial distribution, morphology or function. Inhibition of dMiro has been shown to activate the PAR-1/MARK family kinases, for example, subsequently promoting the pathological phosphorylation of tau [31]. Abnormal phosphorylation and toxicity of tau, a microtubule-associated protein, has been broadly associated with neurodegenerative disorders known as tauopathies [108], including AD [109, 110], frontotemporal dementia [111, 112] and progressive supranuclear palsy [113]. Indeed, activation of the PAR-1/MARK-tau pathway has been demonstrated in animal models of AD in addition to patient samples [114–117] (see Fig. 4). Using transgenic *Drosophila* expressing human tau, Iijima-Ando and colleagues demonstrated that RNAi-mediated dMiro knockdown increased human tau phosphorylation at the AD-related site Ser262, resulting in increased levels of active PAR-1 and enhanced tau-induced neurodegeneration [115]. Furthermore, knockdown of Miro produced late-onset neurodegeneration in the fly brain, an effect that could be suppressed by knockdown of *Drosophila* tau or PAR-1 [115]. Interestingly, the heterozygous Miro mutation (miro[Sd32]) has been previously linked to mitochondrial mislocalisation and the amyloid-β 42 (Aβ42)-induced onset of AD symptoms in an alternate fly model [31]. Although further investigations are required to enhance our understanding of the molecular mechanism underlying the onset of amyloid beta plaques by Miro, these results provide initial evidence for the apparent association between Miro and AD. The essential role of Miros in ATP homeostasis has been described above. While mitochondrial transport in both directions by Miros is based on intracellular calcium sensing, the Miros are also possibly involved in intracellular and intra-mitochondrial calcium sensing in isolation. Although a direct connection between intra-mitochondrial calcium sensing by Miros and neuronal function has not been established, it cannot be completely discounted. Indeed, familial AD has been correlated with increased Ca^2+^ release from ER and elevated levels of calcium [118]. It has been proposed abnormally high Ca^2+^ concentrations over time result in neurons exhibiting AD morphology. Although calcium channel inhibitors have been traditionally considered as therapeutic targets for AD in this respect, it is becoming increasingly apparent that inhibitors and modulators of Ca^2+^ signalling and mitochondrial function are attractive therapeutic targets for AD treatment. Furthermore, knockdown of dMiro has been implicated in late-onset AD in *Drosophila* [115]. It therefore remains to be seen whether Miros can also be potential targets for AD treatment.

## Concluding Remarks

It is becoming increasingly evident that the Miros function as integrated molecular machines that regulate a wide variety of processes, from maintaining normal mitochondrial morphology to mitochondrial transport, in addition to participating in quality control of mitochondria through fission and fusion control [42, 65, 119]. We anticipate that the coming years will see the identification of interacting partners of the Miros to assist regulation of these processes. In such endeavours, proteomic profiling promises to be an important tool for revealing protein-protein interactions mediated through the Miros. The molecular role of the Miros in additional cell processes such as endoplasmic reticulum-mitochondrial complex formation, calcium sensing and neuronal function continues to emerge, whilst the significance of the Miros in developmental and neuronal differentiation processes are yet to be fully established [120–122]. It is clear that the Miros function as unique organelle regulators in ways that have not been observed in any human GTPases previously. Previously targeting the Miros has also been shown to clearly inhibit cell migration in oncogenic cell lines [123]. Targeting the Miros for neurological diseases is rather an attractive option since the structural features of the Miros varies from the traditional Ras-like molecules and allosteric modulators developed against the Miros may prove to be effective therapeutic agents. In order to validate the human Miros as a drug target to modulate Ca^2+^ sensing and neuronal damage it is essential to completely understand the molecular role of individual human Miros. This also includes understanding the intramolecular regulation of full-length protein and intramolecular regulation of full-length protein and molecular conformational changes which will provide a better understanding of mode of regulation and intra molecular interaction capacity. Current overview of Miro function comes from limited information available on Miro’s structural information available for EF hands and c-terminal GTPase domain. Biophysical studies involving Miro has been largely focused on Miros role in mitochondrial transport across neurons and how Miros participate as efficient component of larger molecular assembly with Milton/Traks and Dynein in anterograde and retrograde transports. An elegant biophysical study that investigated the mesenchymal stem cell (MSC) rejuvenation as a therapeutic avenue to combat human disorders determined a compelling role of Miros in intracellular mitochondrial movement from mesenchymal stem cells to epithelial cells [124]. Using mouse models and imaging techniques using various fluorescent probes this study established enhanced Miro1 expression increased mitochondrial donor efficiency. This is quite significant since Miro1 overexpressing MSC is seen to enhance therapeutic effects on various models of lung inflammation and injury and therefore Miro1 overexpression is considered an effective route for various stem cell therapies. It will therefore be useful to explore the possibility of combining various content imaging, TIRF microscopy, and time lapse measurements in specific disease conditions that relate to deficient mitochondrial function to determine the wider role played by human Miros.

Computational studies involving molecular simulations and complete structural modelling will be a valuable addition to improve the insight into the enzymatic capabilities, regulation and macromolecular interactions of Miros for work on phosphate releasing and phosphotransfer enzyme revealed the binding characteristics of EGFR [125]. Moreover, analysing the unique behaviour of the individual full-length human Miro will provide valuable clues on the enzymatic activation and intermolecular interaction properties [126]. Therefore, future work on modelling full-length Miros and analysing folding dynamics will be highly useful for gaining information on dynamic rearrangement of Miros’ cytoplasmic region interaction, EF-hand-based calcium sensing ability and implication of mitochondrial transport in neuronal function. This is also vital to rationalise and develop targeted therapies in the future.
